# The expanded genome of *Hexamita inflata*, a free-living diplomonad

**DOI:** 10.1038/s41597-025-04514-x

**Published:** 2025-02-01

**Authors:** Zeynep Akdeniz, Michal Havelka, Michal Stoklasa, Alejandro Jiménez-González, Vojtěch Žárský, Feifei Xu, Courtney W. Stairs, Jon Jerlström-Hultqvist, Martin Kolísko, Jan Provazník, Staffan Svärd, Jan O. Andersson, Jan Tachezy

**Affiliations:** 1 Department of Cell and Molecular Biology, Uppsala University, Uppsala, Sweden; 2 Department of Parasitology, Charles University, Biotechnology and Biomedicine Centre in Vestec (BIOCEV), Staré Město, Czech Republic; 3 Department of Biology, Lund University, Lund, Sweden; 4 Institute of Parasitology, Biology Centre, Czech Academy of Sciences, České Budějovice, Czech Republic; 5 European Molecular Biology Laboratory (EMBL), Genomics Core Facility, Heidelberg, Germany

**Keywords:** Parasitology, Microbial genetics

## Abstract

Diplomonads are anaerobic, flagellated protists, being part of the Metamonada group of Eukaryotes. Diplomonads either live as endobionts (parasites and commensals) of animals or free-living in low-oxygen environments. Genomic information is available for parasitic diplomonads like *Giardia intestinalis* and *Spironucleus salmonicida*, while little is known about the genomic arrangements of free-living diplomonads. We have generated the first reference genome of a free-living diplomonad, *Hexamita inflata*. The final version of the genome assembly is fragmented (1241 contigs) but substantially larger (142 Mbp) than the parasitic diplomonad genomes (9.8–14.7 Mbp). It encodes 79,341 proteins; 29,874 have functional annotations and 49,467 are hypothetical proteins. Interspersed repeats comprise 34% of the genome (9617 Retroelements, 2676 DNA transposons). The large expansion of protein-encoding capacity and the interspersed repeats are the major reasons for the large genome size. This genome from a free-living diplomonad will be the basis for further studies of the Diplomonadida lineage and the evolution of parasitism-free living style transitions.

## Background & Summary

Microbes dominate the eukaryotic branch of life (protists and fungi, but most microbial eukaryotic diversity is uncultivated, understudied and lacks genomic data^1,2^. The protist genomes studied to date vary greatly in genome size and the factors that determine this genome size are complex and multifaceted and may vary depending on the specific lineage and species^2,3^. Diplomonads are flagellated protists containing anaerobic types of mitochondria-related organelles (MROs) lacking organellar genomes. They commonly possess two nuclei and two sets of flagella with associated cytoskeleton, giving the impression of “doubled” cells, which gave them their name. However, it is important to note, that there are also several known genera belonging to the Diplomonadida, that possess “single” morphology, and these are colloquiality called enteromonads^4^. The Diplomonadida is divided into two main lineages, Giardiinae and Hexamitinae (Supplementary Figure 1A). While members of the Giardiinae lineage use the standard codon usage, members of Hexamitinae uses an alternative genetic code^5^. There are both free-living and endobiotic diplomonads, but all species are believed to be anaerobic aerotolerant (microaerophilic), with the free-living species reported only in reducing environments such as stagnant waters, wastewater treatment plants, and anoxic marine basins. *Hexamita inflata*, in the Hexamitinae lineage, is an example of a free-living diplomonad, found in the sediments of ponds and in the water column of marine basins at depths with low O_2_ tension^6^. Here, we present the first draft genome for *H. inflata*, combining different short and long-read DNA sequencing techniques from a monoxenic culture. Our result shows a large protozoan genome (142 Mbp with nearly 80,000 protein-encoding genes) that will be a valuable resource for further studies of protist genome evolution.

## Methods

### Sample culture and collection

**Fig. 1:**
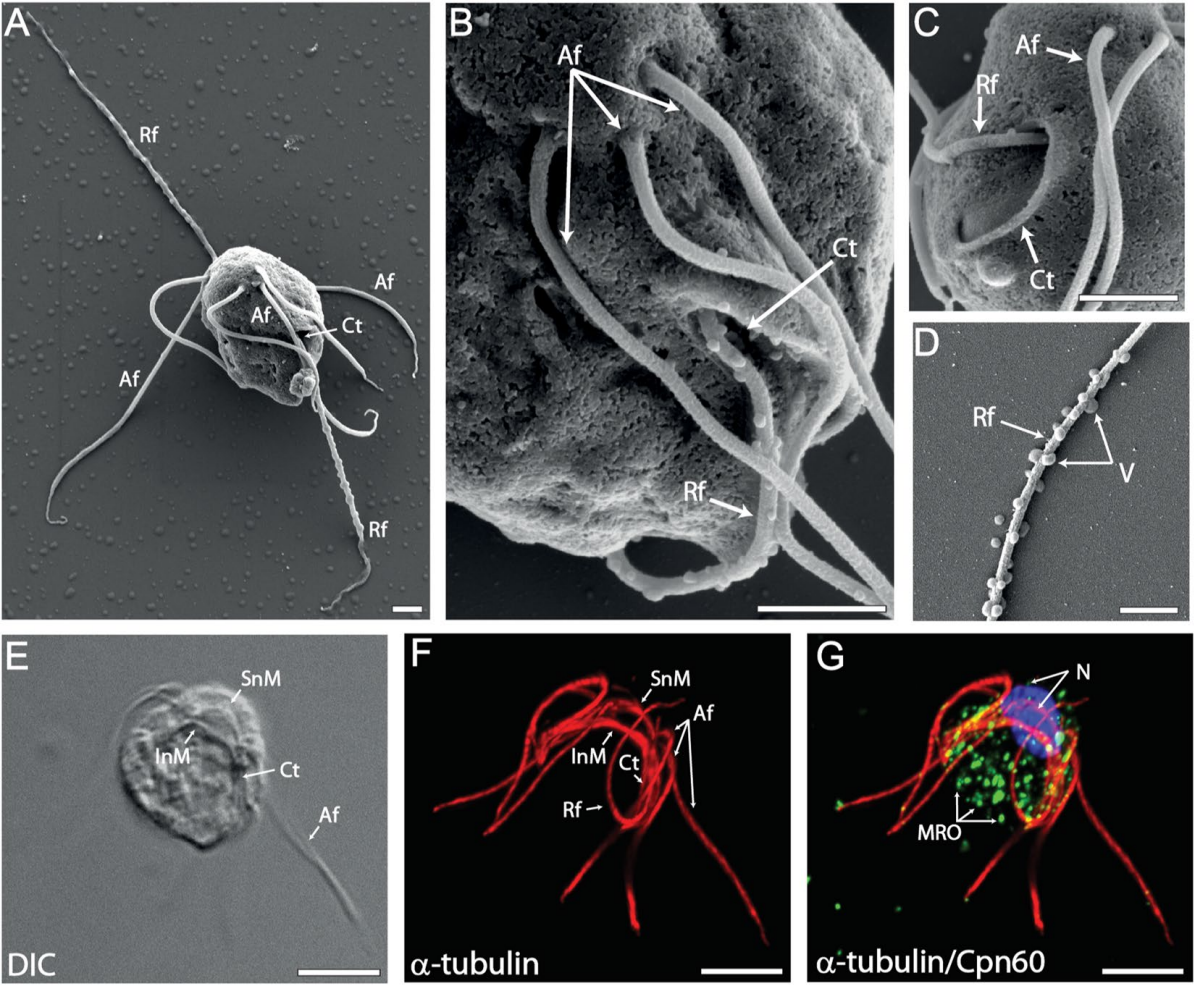
Scanning electron (**A**–**D**) and immunofluorescence micrographs (**E**–**G**) of *H. inflata* trophozoites. **A** – an overview of the cell with two pairs of three anterior (Af) and one recurrent flagellum (Rf). **B** – detailed lateral view of one set of three anterior flagella (Af) and recurrent flagellum (Rf) exiting a cytostome (Ct). **C** – detailed view of the cytostome (Ct) and exiting recurrent flagellum (Rf). **D** – detail of small vesicles (V) on recurrent flagella. **E** – differential interference contrast (DIC) image of *H. inflata* trophozoite. **F** – immunofluorescence microscopy image of microtubular structures of *H. inflata*. Supranuclear bands of microtubules (SnM) cross over the anterior of the two nuclei. Bands of intranuclear microtubules (InM) cross below the two nuclei and extend along the cytostomes (Ct), which each harbour a recurrent flagellum (Rf). **G** – Mitochondria-like organelles (MRO) and microtubular structures. MRO were labelled with anti-Cpn60 antibody (green), and microtubular structures with anti-α-tubulin antibody (red). Nuclei (N) were visualized with DAPI (blue). Scale bars = (**A–D**) 1 μm, (**E,F**) 5 μm.

*Hexamita inflata* (Fig. 1) was isolated from shallow water in a wetland in Řevnice, Czech Republic, by Professor J. Kulda. The organisms were cultivated in 10 ml of modified TYI-S-33 medium (pH 6.8) under semi-anaerobic conditions at 24 °C in Nunclon^TM^ Delta Surface tubes and transferred to fresh media every 5–7 days. The *H. inflata* culture is monoxenic, containing the proteobacterium *Stenotrophomonas maltophilia*^7^. Cryostabilates are deposited in the culture collection at Charles University, BIOCEV, Czech Republic.

### Scanning electron microscopy

To examine the ultrastructure of *H. inflata*, we employed scanning electron microscopy using standard approaches. Briefly, *H. inflata* cells were fixed with 1% formaldehyde in growth media for 30 min, washed with PBS, and attached to silanized glass coverslips. Cells were then fixed with 2.5% (v/v) glutaraldehyde in 0.1 M sodium cacodylate in PBS for 1 h. Fixed cells were washed with 0.1 M sodium cacodylate in PBS and postfixed in 1% (w/v) osmium tetroxide in 0.1 M sodium cacodylate in PBS for 1 h. The cells were washed with distilled water and dehydrated with a graded ethanol series from 35% to absolute ethanol. All steps were carried out at room temperature. Coverslips with cells were critical point dried using Bal-Tec CPD 030 and coated with gold using Bal-Tec SCD 050 sputter coater. Images were captured with a JEOL JSM-IT800 high-resolution scanning electron microscope (JEOL Ltd. Tokyo, Japan). There are in total 8 flagella in *H. inflata* (6 anterior and 2 recurrent, Fig. 1). Figure 1A–C show the placement of the anterior (Af) and recurrent flagella (Rf) and one of the two cytostomes (Ct) of *H. inflata*. One recurrent flagellum, most probably covered by secretory vesicles, can be found in each cytostome, which is used for feeding via phagocytosis (Fig. 1D).

### Immunofluorescence microscopy

To further describe the *H. inflata* morphology, cells were fixed in 1% formaldehyde in growth media and processed as described^8^. Microtubular structures and mitochondria-related organelles were visualized by mouse monoclonal anti-α-tubulin antibody (Sigma-Aldrich) and rabbit polyclonal anti-*Giardia intestinalis* Cpn60 antibody (provided by Robert Hirt, Newcastle University), respectively. Secondary antibodies used were Alexa Fluor 488 goat anti-rabbit and Alexa Fluor 594 goat anti-mouse antibody (Thermo Fisher Scientific, Waltham, USA). Cells were observed with a confocal microscope Leica TCS SP8 WLL SMD-FLIM. Deconvolution of acquired images was performed with Hyugens 19.04 software (Scientific Volume Imaging, Hilversum, Netherlands) and processed using Imaris 9.7.2. Package for Cell Biologists (Bitplane AG, Zurich, Switzerland). Light- (Fig. 1E) and immunofluorescence microscopy analyses (Fig. 1F,G), using antibodies against α-tubulin and Cpn60, showed the relative localization of the flagella, nuclei and MROs in *H. inflata*.

### DNA and RNA sample preparation and sequencing

#### Total DNA and RNA extraction

Total genomic DNA was extracted using Qiagen Blood & Tissue Kit 50 (per manufacturer’s instructions) and further purified using phenol-chloroform extraction and Qiagen Genomic-tip 20/G. The concentration and quality of the extracted DNA was determined by NanoDrop, Bioanalyzer and agarose gel electrophoresis. DNA shearing for Illumina sequencing was performed on a Covaris M220 instrument (Covaris Ltd, Brighton, UK). To achieve the 450-bp fragment size, the Covaris was programmed with the following settings: Duration 35 s, Peak Power 50 W, Duty Factor 20%, 200 Cycles per Burst. The NucleoSpin RNA extraction kit (Roche, Switzerland) was used for total RNA isolation according to the manufacturer’s protocols. RNA purity was verified by Agilent 2100 Bioanalyzer (Agilent Technologies, Santa Clara, USA).

#### DNA and RNA sequencing

Information about genome sequencing, assembly and annotation can be found at European Nucleotide Archive as Project PRJEB61042^9^. DNA sequencing libraries for Illumina sequencing were prepared using the NEBNext® Ultra™ II DNA Library Prep Kit for Illumina (New England Biolabs, Ipswich, MA, USA) according to the manufacturer’s instructions. The quality of the DNA library was assessed using a 2100 Bioanalyzer (Agilent Technologies, Santa Clara, USA). Paired-end sequencing was performed twice, using the MiSeq Re-agent Kit v3 (600 cycles) resp. MiSeq Re-agent Kit v2 (500 cycles) on an Illumina MiSeq platform (Illumina, San Diego, CA, United States) at Charles University, BIOCEV. This generated 45,027,870 cleaned reads^10^ and 12 Gbp sequence (Supplementary Table 1). Total genomic DNA (20 μg), extracted as above, was also sequenced using a PacBio RS II sequencer at the Uppsala Genome Center, the Science for Life Laboratory (Uppsala University). A 10 kbp PacBio library was generated following the standard SMRT bell construction protocol according to the manufacturer recommended protocol. The library was sequenced on 4 SMRT cells of the PacBio RS II instrument using the P6-C4 chemistry, which generated 435,074 reads^11^ corresponding to 3.5 Gbp with an N50 length of 7951 bp (Supplementary Table 1). We complemented this with long-reads from Oxford Nanopore sequencing at the Charles University, BIOCEV, using approximately 400 ng of total DNA to construct sequencing libraries using the 1D Ligation sequencing kit SQK-LSK108 according to the manufacturer’s instructions (Oxford Nanopore Technologies, UK). The prepared libraries were sequenced using the GridION sequencer (Oxford Nanopore Technologies, UK). This generated 323,327 cleaned reads^12^, corresponding to 3.2 Gbp with an average length of 9784 bp (Supplementary Table 1). RNA sequencing was done starting with 4 μg of total RNA. Libraries were prepared using TruSeq Stranded mRNA Library Prep (Illumina) and sequenced as above. A total of 37 M paired Illumina reads (123 bp × 2, Supplementary Table 1) were assembled using Trinity with default settings, yielding 62,069 assembled transcripts^13^.

#### Genome assembly

PacBio high-quality long reads were first used for the initial *de novo* genome assembly using the standard SMRT Analysis (v2.3.0) pipeline. Reads were assembled with HGAP^14^ followed by consensus sequence calling with Quiver (Supplementary Table 2). Quality controlled Illumina reads were subsequently mapped to the PacBio assembly using BWA .0.5.9^15^ and Pilon v1.21^16^ to correct sequencing errors based on the mapped bam file. We used MaSuRCA v3.3.5^17^ with the support of the Flye assembler^18^ making a first hybrid assembly (MaSuRCA_1) combining Illumina reads with PacBio reads (Supplementary Table 2).

One major challenge in genome assembly is the presence of bacteria in the cell culture. There is one major bacterial species in the culture (*S. maltophilia*) with a sequenced genome, which facilitates removal of most bacterial reads, but we used a stringent, circular approach to remove bacterial reads (Supplementary Figure 1B), similar to what we have used in an earlier study^19^. Non-*Hexamita* read detection was done on this assembly by BLASTn v2.8.1 + searches against reference prokaryotes and human genome with the cut-off E value of 1e-10. Non-*Hexamita* reads were also identified by mapping all PacBio and Illumina reads from *H. inflata* against bacterial contigs identified in HGAP assembly via BWA .0.5.9^15^. Only reads considered to be part of *H. inflata* were included in a second assembly using MaSuRCA (MaSuRCA_2, Supplementary Table 2). This resulted in 1689 contigs and 105.5 Mbp in total assembled genome size.

In the next round of assembly (MaSuRCA_3) we also included Nanopore sequencing reads. Reads from all three sequence technologies were mapped to the *non-Hexamita* contigs from the HGAP and MaSuRCA_2 assemblies using BWA .0.5.9 and only non-mapping reads were used as input for MaSuRCa. The MaSuRCA_3 assembly was obtained by using the MaSuRCA assembler with the cleaned PacBio, Illumina, and Nanopore reads (Supplementary Table 2). This resulted in a hybrid assembly with 1241 contigs and 142.6 Mbp in size with a N50 value of 271 Kbp after removal of non-*Hexamita* contigs (Supplementary Table 2). We also analyzed the transcriptome coverage of the different assemblies using the assembled transcripts data (62,069 reads) and 96.7% of the assembled transcripts mapped to the MaSuRCA_3 assembly (Supplementary Table 2). We cleaned the raw reads even further with the three contamination contigs that have been obtained from the MaSuRCA_3 assembly and did a new assembly. However, this resulted in a more fragmented assembly (MaSuRCA_4), smaller total size and fewer mapped reads from RNAseq (Supplementary Table 2). Therefore, the MaSuRCA_3 assembly was selected as the final *H. inflata* genome assembly^20^ and it is considerably larger (142.7 Mbp) than previously studied diplomonad genomes (Table 1).

**Table 1:** Summary statistics for selected Metamonada genomes.

	*H. inflata*	*S. salmonicida*	*G. intestinalis*	*G. muris*	*K. bialata*	*C. membranifera*
**Genome Size (Mbp)**	142.6	14.7	12.6	9.8	51	24.2
**Chromosomes (scaffolds)**	ND (1 241)	9 (42)	5 (38)	5 (59)	ND (11 564)	ND(69)
**Protein encoding Genes**	79 341	8 661	4 963	4 653	17 389	8 300
**G + C content (%)**	33.3	33.5	46.3	54.7	49.4	57.1
**Mean/Median protein size**	352/228	384	635	578/428	333	355/253
**Mean/Median intergenic size**	734/412	527/173	477	264/37	597	1151/199
**Coding density (%)**	58.7	69.5	81.5	84.5	ND	47.9
**Number of introns**	9	4 cis	8 cis, 5 trans	3 cis, 5 trans	124 912	ND

### Gene prediction and functional annotation

#### Structural annotation

We developed a custom pipeline for *ab initio* gene prediction in the *H. inflata* genome. Prokaryotic and eukaryotic gene prediction software are inappropriate for gene calling in diplomonads because they are eukaryotic organisms with very few known introns^21^, and *H. inflata* uses an alternative genetic code^5^. Therefore, we combined the results from two gene prediction software packages: GlimmerHMM v3.0.1^22^ and Prodigal v2.50^23^. We first used protein-coding genes from *Spironucleus salmonicida*^24^ to search against the gene models predicted by Prodigal. Using BLASTp, we assembled a training set of genes for GlimmerHMM. This yielded a training set with 1776 trusted gene models with e-values lower than 1e-10, start and stop regions within 10% of the sequence area as well as sequence length longer than 150 amino acids (aa). Additionally, nine intron-containing genes (Supplementary Table 3) were manually added to the training file. Intron-containing genes were found by tBLASTn and BLASTn searches of known intron-containing genes using *G. muris, G. intestinalis*, and *S. salmonicida*^21^ as queries against the *H. inflata* assembly. GlimmerHMM is efficient in predicting single-exon genes, it can be trained using known introns and we have successfully used GlimmerHMM in our previous genome studies involving diplomonads^24,25^. We thus included all unique GlimmerHMM predictions in our preliminary structural annotation. This result was combined with Prodigal predictions to improve the quality of the structural annotation.

Prodigal and GlimmerHMM yielded 122,331 and 101,914 gene models, respectively. Of these, 74,196 were identical, sharing the same start and stop codon. Furthermore, 693 out of 48,135 gene predictions from Prodigal, which were non-identical to GlimmerHMM predictions, had a sequence similarity match against the *nr* database with an e-value lower than 1e-5 using BLASTp. One hundred thirty of these were found to not overlap with any gene predicted by GlimmerHMM, so they were added to the structural annotation. A final comparison and clean out of the gene models resulted in 101,051 gene models in the structural annotation.

#### Functional annotation

The functional annotation used information from several sources in a hierarchical manner. First, we performed a similarity search against the known diplomonad genomes (*G. intestinalis*^26^*, G. muris*^25^*, S. salmonicida*^24^) with Diamond (v2.0.7)^27^ in ultra-sensitive mode and BLASTp (using an e-value of 1e-5). Subsequently, the *Trepomonas* sp. PC1^19^ transcriptome was used, and finally the *nr* database. Genes that could not be assigned a function using this approach were annotated with eggNOG-mapper v1.0.3^28^ and InterProScan 5.47^29^, respectively. We found that 42,810 out of a total of 101,051 gene models were functionally annotated, whereas the remaining 36,351 genes were annotated as hypothetical. 21,710 of the hypothetical genes were shorter than 100 aa and were discarded. In total, 79,341 protein encoding genes were retained in the final version of the genome and used for further analyses (Table 1). The assembled set of RNAseq reads were used to support the protein encoding genes and to define start codons. The *H. inflata* proteome in FASTA format can be down-loaded at 10.6084/m9.figshare.26298478.v1.

#### Analyses of the expanded protein repertoire in *H. inflata*

The *H. inflata* genome is considerably larger, 142.7 Mbp, than most of the previously studied Metamonada genomes (Table 1). To test if this increase in genome size is accounted for by an increased number of introns – as has been reported in other free-living metamonads (*K. bialata* with 124,912 introns, Table 1), we first used known introns from diplomonads^21^ and screened their presence in the *H. inflata* genome as described above. This revealed 9 introns in two genes, ribosomal proteins L7 and S24 (Supplementary Table 3). We also mapped our assembled RNASeq reads against the *H. inflata* genome using BLASTn with permissive criteria. However, no clear novel candidates of introns were identified using this approach. All introns have probably not been identified in *H. inflata*, more specific search approaches are needed, but our data suggest that the *H. inflata* genome is intron-poor, like most studied diplomonads (Table 1). We also examined the number of tRNA genes, as tRNA gene expansions in tandem arrays have been found to be associated with genome expansions in other protozoans, like the intestinal parasite *Entamoeba histolytica*^30^. We identified 840 tRNA genes in *H. inflata*, using tRNAScan-SE 2.0^31^ but the relative number of tRNA genes compared to genome size is similar to *G. intestinalis*^26^ and *S. salmonicida*^24^ so this feature cannot by itself explain the genome expansion observed in *H. inflata*. The rDNA genes are expanded in repeated regions in the genome of *G. muris*^25^, making up 2% of the genome, whereas the genome otherwise is stream-lined. Using RoboDetector^32^ we could show that the relative number of rDNA genes in *H. inflata* is similar to *G. intestinalis* and *S. salmonicida* (Supplementary Table 4) but not expanded to the same degree as in *G. muris* (only 0.15% of the *H. inflata* genome, Supplementary Table 4).

**Fig. 2:**
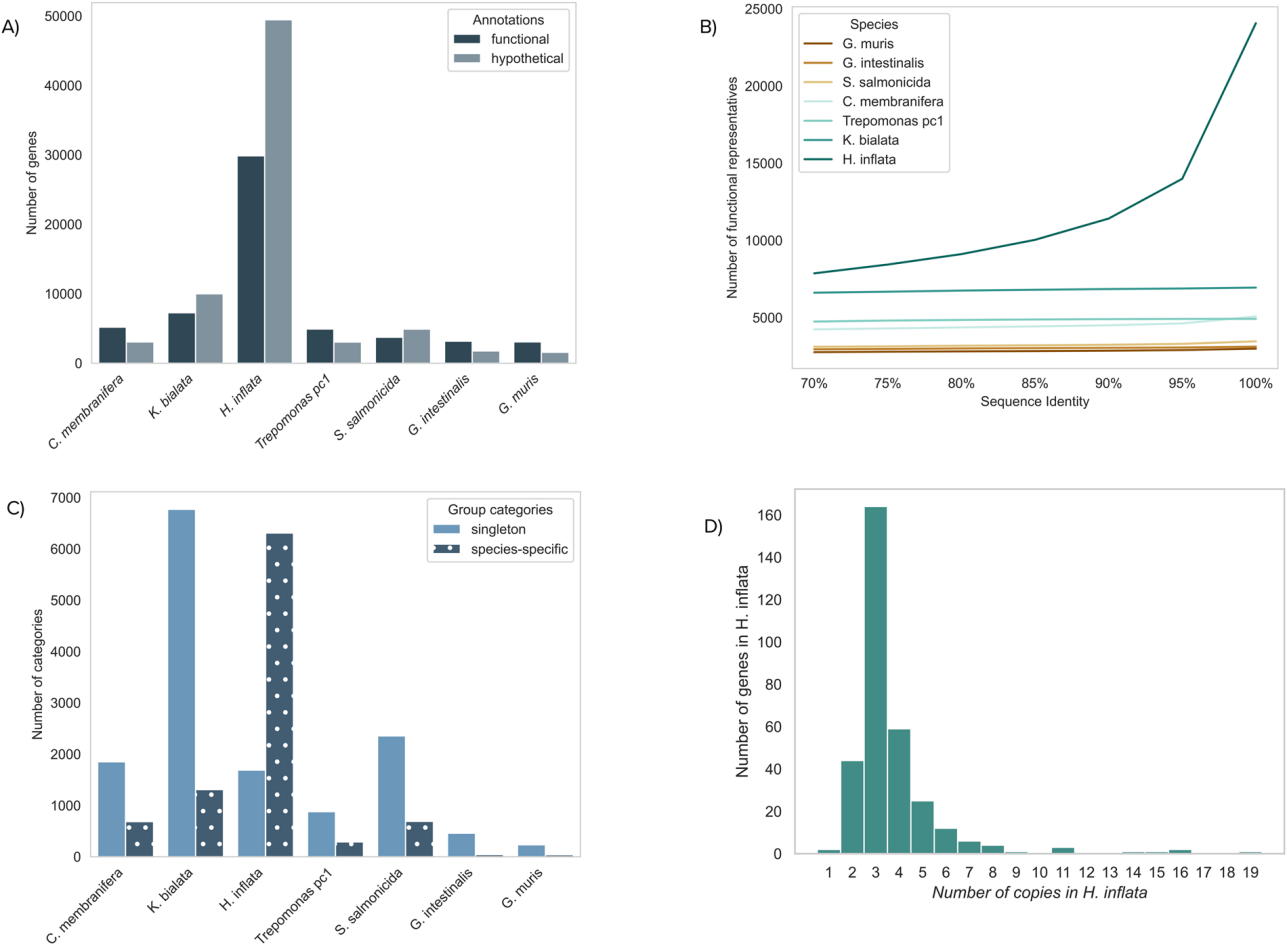
Analyses of the *H. inflata* proteome. (**A**) Categorization of functional and hypothetical proteins annotated by InterProScan from six Metamonada genomes and one transcriptome (*Trepomonas PC1*). (**B**) A line plot quantifying the clustering of the Metamonada genomes and transcriptomes, with functional proteins grouped at incrementally increasing sequence similarity thresholds. (**C**) Visualization of species-specific cluster categories.Singletons are represented as unique single-copy genes within a species. Species-specific orthogroups comprising genes only from one species are depicted as dotted bars. (**D**) The copy number distribution of *H. inflata* genes, belonging to orthogroups which exist as single copies in other Metamonada species.

The number of protein-encoding genes is much higher in *H. inflata* than in other diplomonads (79,342 proteins compared to 4,963 in *Giardia* and 8,661 in *S. salmonicida*, Table 1). The average length of a protein in *H. inflata* is 352 aa (Table 1), well within the range of other Metamonada genomes (333–635 aa^24–26^). We observed a larger mean intergenic size between protein encoding genes in *H. inflata*, 734 bp, compared to 470 bp in *G. intestinalis* and 460 bp in *S. salmonicida* (Table 1). Thus, the expanded protein repertoire combined with putative regulatory regions is a major component of the expanded genome of *H. inflata*. However, it is not clear if this increase is reflected in a larger number of proteins with different functional annotation. To study this, we compared the ratio of genes with functional annotations and genes encoding hypothetical proteins for seven Metamonada species (Fig. 2). All the genomes/transcriptomes were re-annotated by InterProScan^29^ using default parameters and genes that could not be associated with any known function were registered as hypothetical proteins (Fig. 2A). In *H. inflata*, 29,874 genes have functional annotations, and 49,467 are hypothetical proteins. Out of the 49,467 hypothetical proteins, 14,945 (30%) are found in at least one of the other metamonads and are therefore annotated as conserved hypothetical proteins. These analyses show that *H. inflata* has much more proteins with functional annotations, compared to other metamonads (Fig. 2A). One possible explanation for this increase in protein-encoding capacity could be recent whole-genome amplifications in the *H. inflata* genome. To test this hypothesis, we grouped the proteins in the Metamonada genomes by gradually increasing sequence identity thresholds using Cd-Hit^33^. This analysis indeed suggests that *H. inflata* has had more recent genome amplifications than the rest of the Metamonada genomes since a large part of the protein-encoding genes show more than 95% identity at the protein level, resulting in a large reduction of functional clusters when the identity is gradually reduced from 100% to 70% (Fig. 2B). In contrast, the other diplomonads do not show the same level of reduction on functional clusters, going from 100% to 70% identity (Fig. 2B). We also used the BUSCO^34^ software (v5) to assess the genome quality and protein duplications (Supplementary Figure 2). This shows that the completeness of the *H. inflata* genome is similar to the completeness in previously sequenced diplomonad genomes and also that many of the eukaryotic proteins used to evaluate the completeness are duplicated, which is not seen in the other diplomonad genomes (Supplementary Figure 2). The low level of fragmented genes is similar to other diplomonads with few introns, as compared to the intron-rich *K. bialata* genome (Supplementary Figure 2). It should be noted here that BUSCO is not an optimal tool in Metamonads since many of the conserved proteins used in the BUSCO analyses have been lost^35^. To further explore the *H. inflata* proliferation in the protein space, we compared and grouped proteins in six chosen Metamonada genomes (*G. intestinalis*^26^*, G. muris*^25^, and *S. salmonicida*^24^*, Kipferlia bialata*^36^ and *Carpediemonas membranifera*^35^) and the *Trepomonas sp. PC1*^19^ transcriptome using Orthofinder v2.5.2^37^. This produced gene clusters, with each cluster representing an orthogroup (OG) comprised of orthologous genes from diverse species (Supplementary Table 5). Conversely, when a cluster consisted of protein sequences originating solely from one species, we classified it as a species-specific OG (Fig. 2C). Additionally, genes identified as singletons^38^ (genes that do not fall into any OGs in any of the 7 species and are highly divergent) are also usually incorporated into the species-specific gene category (Fig. 2C). The analysis clearly showed that the number of species-specific genes is higher in *H. inflata* (6311 OGs and 1690 singletons, representing 53,308 genes), compared to the other species (Fig. 2C and Supplementary Table 5). The most expanded protein superfamilies are Leucine-rich-repeat and Homeobox proteins, similar to what was seen in the transcriptome of another free-living diplomonad, *Trepomonas*^19^. Further analyses can reveal how environments affect the evolution of protein and multi-gene families in parasitic and free-living diplomonads. We identified 325 genes that were single-copy genes in the other Metamonads included in the study (Supplementary Table 6). Only 2 of these (0.6%) are found as a single-copy gene in *H. inflata*, the rest are found in two or more copies (Fig. 2D). This analysis suggests the amplification of ancestrally existing genes has occurred extensively in *H. inflata*, partially accounting for the expanded genome and the larger number of protein-coding genes.

#### Interspersed repeat regions

**Fig. 3:**
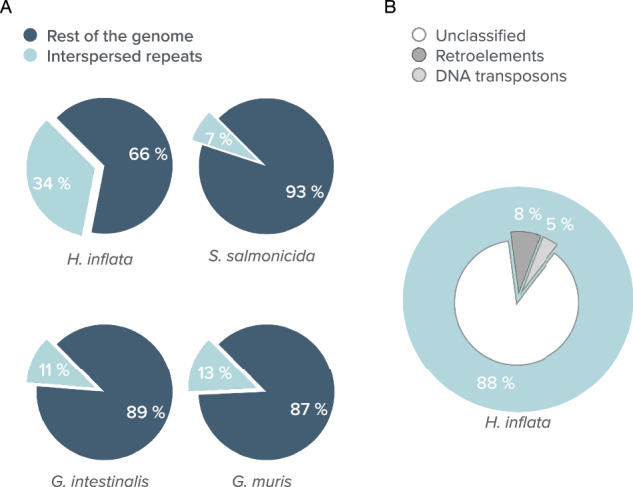
Composition of Interspersed repeats in diplomonad Genomes and Transposable Element Content in *H. inflata*. (**A**) The fraction of protein-coding genes (in base pairs) and repeat elements present in the diplomonad genomes is depicted. (**B**) The content of annotated interspersed repeat elements within the *H. inflata* genome.

RepeatMasker 4.1.5 was used to study the level of repeat regions in the genomes of *H. inflata*, *G. intestinalis*, *G. muris* and *S. salmonicida* (Fig. 3). It showed that the genomes of the different diplomonads exhibit varying compositions of interspersed repeats within their genomes. For instance, the *H. inflata* genome is constituted of 34% interspersed repeats (Fig. 3), while the equivalent figures for the parasitic diplomonads range from 7–13% (Fig. 3A). Interestingly, the majority of repeat elements in parasitic diplomonads remain uncharacterized. The *G. intestinalis* contain 74 long interspersed nuclear elements (LINEs), whereas *S. salmonicida* has 29 DNA transposons in the analysis. 13.4% of the *G. muris* genome consists of unidentified interspersed repeats, while 5.17% of the genome is composed of small RNA. The *H. inflata*’s genome shows further complexity, with 2676 DNA transposons and 9617 retrotransposons including LINEs (8606) and 727 long terminal repeat (LTR) elements (Fig. 3B).

To conclude, the large genome size of *H. inflata*, compared to other diplomonads, can be explained by a combination of proliferation of repeated regions, random duplication of all parts of the genome including genes for stable RNA and proteins, and an even larger expansion of the protein repertoire.

## Data Records

This Whole Genome Shotgun project has been deposited at DDBJ/ENA/GenBank under project number PRJEB61042^9^. The genome assembly described in this paper has ENA assembly accession GCA_963988835.2^20^. Raw DNA sequence reads are deposited at ENA: PacBio^11^, Illumina^10^ and Nanopore^12^ reads with accession numbers ERX10822065, ERX10822047 and ERX10822163, respectively. Assembled RNASeq reads can be found at Figshare^13^.

## Technical Validation

The main challenge in assembling the *H. inflata* genome is the presence of *S. maltophilia* reads, the alternative genetic code and the large and repeated nature of the *H. inflata* genome. This means that a very strict procedure of stepwise contamination cleaning and assembly has to be followed, combined with the mapping of RNAseq reads to the assembled genome. Our stepwise technical validation is shown in Supplementary Table 2. It shows how the assembly improves step by step during the assembly using different data sets and contaminating bacterial reads are removed from the final assemblies. Mapping of RNAseq reads improves from 83.7% in the first HGAP assembly to 96.7% in the best assembly (MaSuRCA3, Supplementary Table 2).

## Supplementary information

Supplementary Figure 1

Supplementary Figure 2

Supplementary Table 1

Supplementary Table 2

Supplementary Table 3

Supplementary Table 4

Supplementary Table 5

Supplementary Table 6

## Data Availability

The versions of the software employed in this study have been specified in the Methods section. The default parameter was used, if no parameter was provided.
