# Supplementary figures and images for: The expanded genome of Hexamita inflata, a free-living diplomonad
Source: Sci Data. 2025 Feb 1;12:192. doi: 10.1038/s41597-025-04514-x (PMC11787283; doi:10.1038/s41597-025-04514-x)

Supplementary Figure 1

## A. Species tree

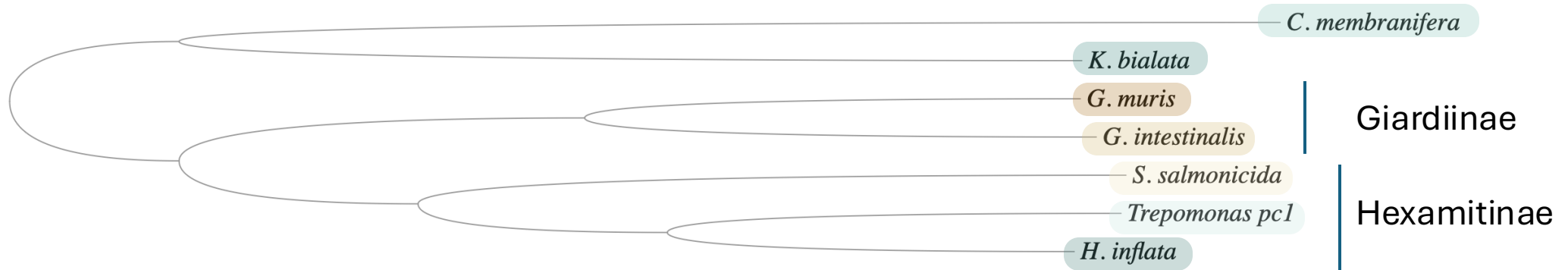

## B. Bacterial contig contamination removal

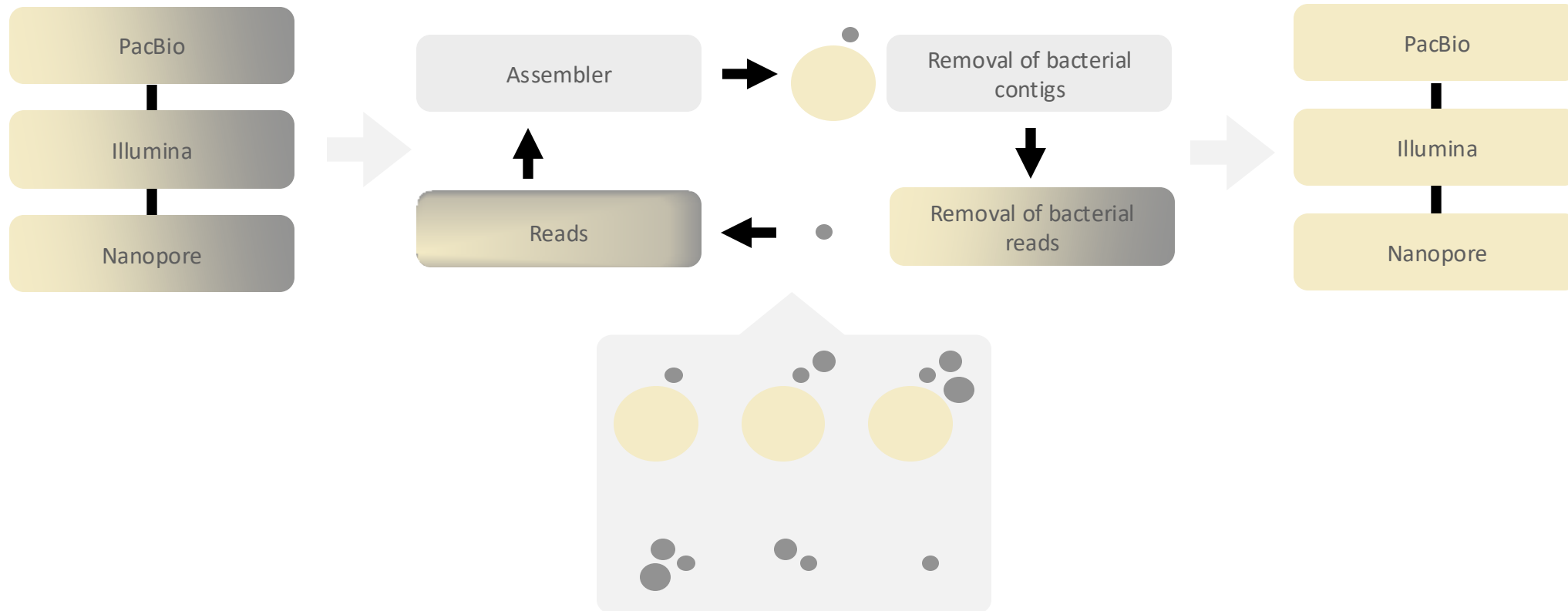

Supplement: Supplementary file 1 — Supplementary Figure 1 [file 41597_2025_4514_MOESM1_ESM.pdf]

# BUSCO Assessment Results

Busco  
Complete genes

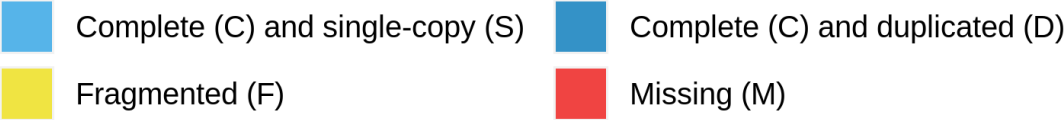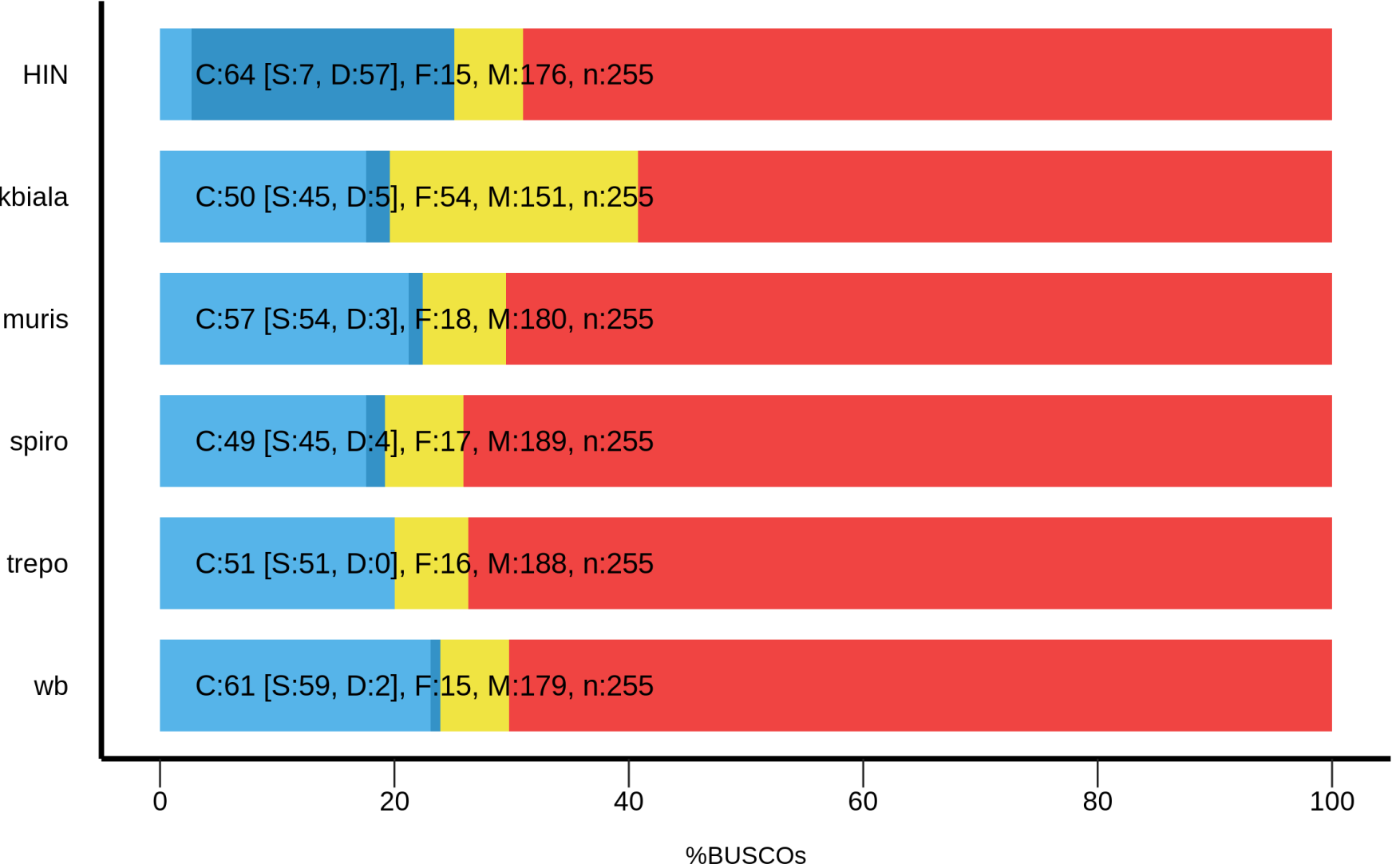

Supplement: Supplementary file 2 — Supplementary Figure 2 [file 41597_2025_4514_MOESM2_ESM.pdf]
